# Driver Mutations of Pancreatic Cancer Affect Ca^2+^ Signaling and ATP Production

**DOI:** 10.1093/function/zqad035

**Published:** 2023-07-04

**Authors:** Kinga B Stopa, Filip Łoziński, Agnieszka A Kusiak, Jacek Litewka, Daria Krzysztofik, Sylwester Mosiołek, Jan Morys, Paweł E Ferdek, Monika A Jakubowska

**Affiliations:** Malopolska Centre of Biotechnology, Jagiellonian University, ul. Gronostajowa 7A, 30-387 Kraków, Poland; Doctoral School of Exact and Biological Sciences, Jagiellonian University, ul. Łojasiewicza 11, 30-348 Kraków, Poland; Malopolska Centre of Biotechnology, Jagiellonian University, ul. Gronostajowa 7A, 30-387 Kraków, Poland; Department of Medical Biotechnology, Faculty of Biochemistry, Biophysics and Biotechnology, Jagiellonian University, ul. Gronostajowa 7, 30-387 Kraków, Poland; Doctoral School of Exact and Biological Sciences, Jagiellonian University, ul. Łojasiewicza 11, 30-348 Kraków, Poland; Department of Cell Biology, Faculty of Biochemistry, Biophysics and Biotechnology, Jagiellonian University, ul. Gronostajowa 7, 30-387 Kraków, Poland; Doctoral School of Exact and Biological Sciences, Jagiellonian University, ul. Łojasiewicza 11, 30-348 Kraków, Poland; Department of Cell Biology, Faculty of Biochemistry, Biophysics and Biotechnology, Jagiellonian University, ul. Gronostajowa 7, 30-387 Kraków, Poland; Malopolska Centre of Biotechnology, Jagiellonian University, ul. Gronostajowa 7A, 30-387 Kraków, Poland; Doctoral School of Exact and Biological Sciences, Jagiellonian University, ul. Łojasiewicza 11, 30-348 Kraków, Poland; Malopolska Centre of Biotechnology, Jagiellonian University, ul. Gronostajowa 7A, 30-387 Kraków, Poland; Department of Medical Biotechnology, Faculty of Biochemistry, Biophysics and Biotechnology, Jagiellonian University, ul. Gronostajowa 7, 30-387 Kraków, Poland; Malopolska Centre of Biotechnology, Jagiellonian University, ul. Gronostajowa 7A, 30-387 Kraków, Poland; Department of Medical Biotechnology, Faculty of Biochemistry, Biophysics and Biotechnology, Jagiellonian University, ul. Gronostajowa 7, 30-387 Kraków, Poland; Department of Cell Biology, Faculty of Biochemistry, Biophysics and Biotechnology, Jagiellonian University, ul. Gronostajowa 7, 30-387 Kraków, Poland; Malopolska Centre of Biotechnology, Jagiellonian University, ul. Gronostajowa 7A, 30-387 Kraków, Poland

**Keywords:** Ca^2+^ signaling, driver mutations, KPC mouse model, Kras, pancreatic acinar cell, pancreatic cancer, pancreatic duct cell, Trp53

## Abstract

Glandular pancreatic epithelia of the acinar or ductal phenotype may seem terminally differentiated, but they are characterized by remarkable cell plasticity. Stress-induced trans-differentiation of these cells has been implicated in the mechanisms of carcinogenesis. Current consensus links pancreatic ductal adenocarcinoma with onco-transformation of ductal epithelia, but under the presence of driver mutations in *Kras* and *Trp53*, also with trans-differentiation of pancreatic acini. However, we do not know when, in the course of cancer progression, physiological functions are lost by mutant acinar cells, nor can we assess their capacity for the production of pancreatic juice components. Here, we investigated whether two mutations—Kras^G12D^ and Trp53^R172H^—present simultaneously in acinar cells of KPC mice (model of oncogenesis) influence cytosolic Ca^2+^ signals. Since Ca^2+^ signals control the cellular handling of digestive hydrolases, any changes that affect intracellular signaling events and cell bioenergetics might have an impact on the physiology of the pancreas. Our results showed that physiological doses of acetylcholine evoked less regular Ca^2+^ oscillations in KPC acinar cells compared to the control, whereas responses to supramaximal concentrations were markedly reduced. Menadione elicited Ca^2+^ signals of different frequencies in KPC cells compared to control cells. Finally, Ca^2+^ extrusion rates were significantly inhibited in KPC cells, likely due to the lower basal respiration and ATP production. Cumulatively, these findings suggest that driver mutations affect the signaling capacity of pancreatic acinar cells even before the changes in the epithelial cell morphology become apparent.

## Graphical Abstract

**Graphical Abstract:**
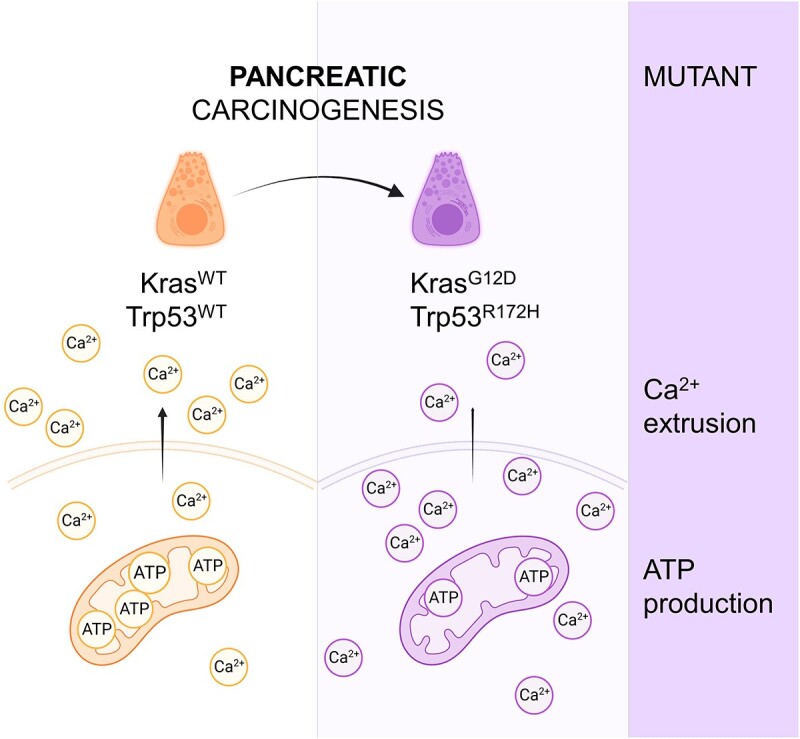

## Introduction

**Figure 1.**
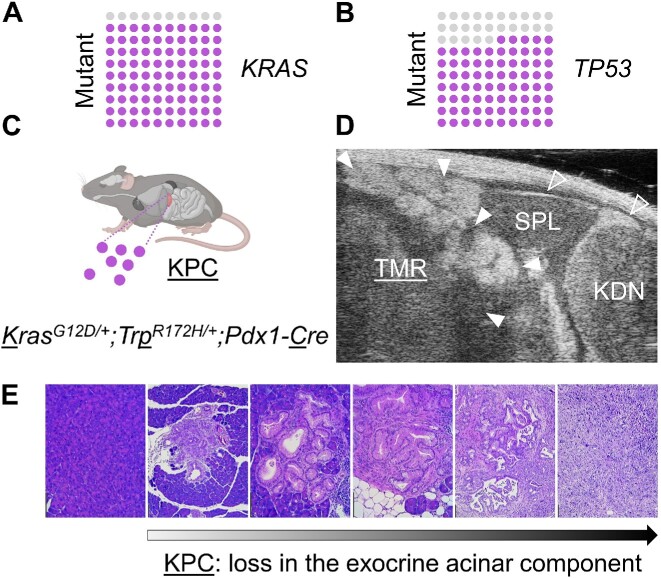
Genetic model of pancreatic carcinogenesis. Pancreatic carcinogenesis in humans is induced by the presence of genetic driver mutations: (A) *KRAS* gene is mutated in 90% of the patients and (B) *TP53* is mutated in 75% of the cases. (C) KPC mouse (Kras^G12D/+^;Trp53^R172H/+^;Pdx1-Cre): This genetic model of carcinogenesis develops pancreatic neoplasias owing to Cre-induced expression of mutant Kras^G12D^ and mutant Trp53^R172H^ in pancreatic epithelia.^12^ Mosaic expression of Cre recombinase under control of Pdx1 transcription factor in the exocrine pancreatic component simultaneously activates oncogenic Kras^G12D^ and causes a loss in Trp53 suppressor functions, resulting in cancer development and progression, also to distant metastatic sites. (D) Representative ultrasound image of KPC mouse abdomen, showing the development of a solid tumor in the pancreas. TMR: tumor; SPL: spleen; KDN: kidney; open arrowheads: organs; closed arrowheads: pancreatic tumor. The image was collected in B-mode, general imaging mode, using the following parameters of signal collection: frequency 40 MHz, power 100%, gain 22 dB, depth 10.00 mm, and width 14.08 mm; and signal display: dynamic range 65 dB, display map G5, brightness 50, and contrast 50. (E) Histological hematoxylin and eosin staining of mouse pancreatic tissues collected from KPC mice at different stages of pancreatic carcinogenesis. As the disease progresses, the normal exocrine pancreatic component is lost (in mice >3-month-old) and becomes gradually replaced by (pre)malignant epithelial structures, and finally with cancerous tissues of 100% organ penetration (mice 6- to 9-month-old). Scale bars: 100 μm.

Pancreatic cancer (PC) is one of the most lethal malignancies, with an overall survival rate below 11%.^1^ Due to the accumulation of genetic changes, for example, the presence of “driver mutations” in *KRAS* and *TP53* genes (Figure 1), the exocrine component of the pancreas becomes transformed and loses its functions.^2,3^ This irreversibly affects the physiology of the exocrine pancreas, including the production, storage, and release of digestive enzymes.

As counterintuitive as it may seem, genetically altered epithelia of the pancreatic ducts are not the exclusive source of cellular precursors for the most common form of PC: pancreatic ductal adenocarcinoma (PDAC). Studies on the phenotypic transition and its role in pancreatic carcinogenesis revealed a considerable capacity of pancreatic acinar cells (PACs) to undergo morphogenetic changes and, as a result, acquire a duct cell-like phenotype. The consensus has been established that PC arises equally often from the acinar and ductal epithelia.^4^ In particular, the multistep cell trans-differentiation processes yield a number of intermediate stages between these two cell types,^5^ further contributing to disease progression and the intratumoral phenotypic heterogeneity in PC^6^ (Figure 1).

The Kras-driven malignant transformation not only perpetuates pancreatic carcinogenesis but also induces clonal epithelial loss of function (Figure 1), thus reducing the effective volume of cells capable of hydrolase secretion and production of pancreatic juice. At the early stages of PC development, faulty digestion (often manifesting as diarrhea or constipation)^7^ rarely alerts patients who may simply ignore symptoms of a “clinically silent” disease in the pancreas. However, the pathophysiological mechanisms that drive the transition of normal epithelial cells to the premalignant stages of pancreatic carcinogenesis are not well understood.

In normal pancreatic epithelia, the secretion of digestive hydrolases by acinar cells occurs via exocytosis concurrent with the secretion of Ca^2+^-rich fluid.^8–10^ Tightly regulated spatiotemporal oscillations (regular waves) of Ca^2+^ serve as a physiological signal for enzyme release from acinar epithelial cells. However, when these signals are dysregulated^10^ and escalate into global and sustained Ca^2+^ elevations, digestive enzymes become activated prematurely in situ in PACs,^10^ causing necrosis and the release of damage-associated molecular patterns (DAMPs) from injured and dead cells.^11^ Surprisingly, the role of pathophysiological Ca^2+^ signals is much less evident in the development of PC, compared to other pancreatic pathologies also characterized by the inflammatory background, such as acute pancreatitis (AP).

This has prompted us to study cytosolic Ca^2+^ signals in PACs isolated from KPC (Kras^G12D/+^;Trp53^R172H/+^;Pdx-1-Cre) mice, a genetic animal model of pancreatic carcinogenesis.^12^ Owing to the presence of mutations in single alleles of the *Kras* and *Trp53* genes under the control of the Pdx1-Cre construct, KPC mice recapitulate a multistep (premalignant to cancer) model of PC development (Figure 1). Using KPC and control-Cre PACs, we first compared Ca^2+^ signals elicited by low physiological doses of acetylcholine (ACh) that in normal PACs evoke cytosolic Ca^2+^ oscillation. In another set of experiments, we applied menadione (Men) that elicits a more irregular pattern of intracellular Ca^2+^ elevations in PACs: oscillations, calcium transients, formation of the cytosolic Ca^2+^ plateau, or a combination of all of the above. A detailed numerical analysis of the area under response curves, as well as a direct comparison of the proportions of different types of cytosolic Ca^2+^ signal patterns, shows that, on average, Ca^2+^ elevations in KPC cells were more prominent and prolonged compared to controls (with Cre, WT Kras, and WT Trp53). The application of a Ca^2+^ extrusion protocol revealed a significantly slower Ca^2+^ efflux from the stores in KPC PACs compared to control-Cre cells, but the expression levels of the plasma membrane Ca^2+^ ATPase (PMCA) remained unchanged. Finally, the analysis of the cellular energy metabolism showed decreased mitochondrial respiration in KPC cells compared to normal PACs. Collectively, these findings suggest an impaired Ca^2+^ handling in premalignant secretory epithelium expressing Kras^G12D^ and Trp53^R172H^, even when no morphological changes are yet evident in PACs.

## Materials and Methods

### Selected Chemicals and Consumables

2-[4-(2-Hydroxyethyl)piperazin-1-yl]ethane-1-sulfonic acid (HEPES), 3,12-Bis(carboxymethyl)-6,9-dioxa-3,12-diazatetradecane-1,14-dioic acid (EGTA), 3-(morpholin-4-yl)propane-1-sulfonic acid (MOPS), acetylcholine, anti-vinculin mouse monoclonal antibody (#V9131), bicinchoninic acid (BCA), calcium chloride, carbonyl cyanide-4 (trifluoromethoxy) phenylhydrazone (FCCP), collagenase type IV, dimethyl sulfoxide (DMSO), eosin with phloxine, ethanol, hematoxylin, Immobilon Western Chemiluminescent HRP Substrate, magnesium chloride, menadione, oligomycin, potassium chloride, radioimmunoprecipitation assay buffer (RIPA), rotenone with antimycin A, sodium chloride, Tris-buffered saline (TBS), and Tween 20 were purchased from Sigma-Aldrich/Merck, USA; Agarose, DNA Perfect Leader, and SimplySafe loading dye were purchased from EURx, Poland; Amersham Hybond 0.2 PVDF blotting membranes were purchased from Cytiva, UK; anti-calcium pump pan PMCA ATPase mouse monoclonal antibody (#ab2825) was purchased from Abcam, UK; CellTiter-Glo 3D Cell Viability Assay was purchased from Promega, USA; cOmplete mini, EDTA-free protease inhibitor cocktail, and KAPA mouse genotyping mix were purchased from Roche Diagnostics GmbH, Germany; distilled water, Genomic Mini isolation kit, and Tris buffer were purchased from A&A Biotechnology, Poland; Fluo-4 AM, Hoechst   33258, NuPAGE 4%-12% Bis-Tris Gels, and Nunc 96 Well White/Clear Bottom Plates, TC Surface were purchased from Thermo Fisher Scientific, USA; glucose, glutamine, pyruvate, Seahorse XF Cell Mito Stress Test Kit, and XF DMEM Medium pH 7.4, 5 m m HEPES, phenol red free, were purchased from Agilent, USA; horse anti-mouse IgG Antibody (H + L), peroxidase (#PI-2000), was purchased from Vector, USA; Matrigel, growth factor reduced, phenol red-free, was purchased from Corning, USA; NucView 488 & RedDot 2 Apoptosis and Necrosis Kit was purchased from Biotium, USA; TAE buffer was purchased from Bio-Rad, USA; and Thapsiargin was purchased from Cayman, USA.

### Isolation of Genomic DNA

5 mm tail tip samples were collected from young animals after weaning. DNA was extracted using a Genomic Mini isolation kit, eluted in Tris buffer, and stored for a long term at −20°C.

### Polymerase Chain Reaction

The animal genotype was confirmed by polymerase chain reaction (PCR) using DNA extracted from the tail tips and specific primers under the conditions described in Table 1. PCR was carried out in a ProFlex thermal cycler (Thermo Fisher Scientific), in 0.2 mL plastic tube strips containing 3 μL of genomic DNA, 2.63 μL of KAPA, 0.625 μL of 10 μM primer mix, and 6.25 μL of distilled water.

**Table 1.** Primer Sequences Used for Genotyping, and PCR Reaction Conditions

Gene	Sequence
***Kras* Kirsten rat sarcoma viral oncogene homolog**	
*G12D mutant*	AGC TAG CCA CCA TGG CTT GAG TAA GTC TGC A
*Universal*	CCT TTA CAA GCG CAC GCA GAC TGT AGA
*Wild type*	GTC GAC AAG CTC ATG CGG GTG
**Conditions**	D: 95°C, 2:30 min; A (×34): 95°C, 0:30 min; 68°C, 0:30 min; 72°C, 1 min; E: 72°C, 7 min; 4°C, ∝
**Product sizes**	Wild type: 500 bp only; Mutant: 500 bp and 600 bp
***Trp53* Transformation-related protein 53**	
*R172H Mutant*	F: AGC TAG CCA CCA TGG CTT GAG TAA GTC TGC A R: CTT GGA GAC ATA GCC ACA CTG
*Wild type*	F: TTA CAC ATC CAG CCT CTG TGG
**Conditions**	D: 95°C, 2:30 min; A (×34): 95°C, 0:20 min; 62°C, 0:30 min; 72°C, 0:30 min; E: 72°C, 5 min; 4°C, ∝
**Product sizes**	Wild type: 170 bp only; Mutant: 170 bp and 280 bp
***Pdx1-Cre***	
	F: CTG GAC TAC ATC TTG AGT TGC R: GGT GTA CGG TCA GTA AAT TTG
**Conditions**	D: 95°C, 2:30 min; A (×34): 95°C, 0:30 min; 55°C, 0:30 min; 72°C, 1 min; E: 72°C, 7 min; 4°C, ∝
**Product size**	500 bp, or no product

D: denaturation; A: annealing; E: extension, F: forward; R: reverse.

### Gel Electrophoresis of PCR Products

For the separation of PCR products (1%: *Kras* and *Pdx1-Cre*; 3%: *Trp53*, respectively), 1% or 3% agarose gels were made by dissolving agarose in TAE buffer. SimplySafe loading dye was then added to allow DNA detection in agarose gels. Casted gels were positioned in a Sub-Cell GT Horizontal Electrophoresis System (Bio-Rad, USA), covered with TAE buffer, loaded with 10 μL of DNA or 3 μL of DNA Perfect Leader, and the samples were run at 90 V for 30-60 min. The samples were visualized using a ChemiDoc XRS + Imaging System (Bio-Rad, USA), and the genotypes were determined based on product sizes given in Table 1.

### Experimental Animals

The KPC (LSL-Kras^G12D/+^; LSL-Trp53^R172H/+^; Pdx1-Cre)^12^ mouse breeding pair (mixed background) was transferred from Cardiff University to the Jagiellonian University institutional animal units in 2018. The animals were kept in a 12 h light/dark regimen, in individually ventilated cages (up to 5 mice) with aspen wood bedding material and environmental enrichment, and free access to food (standard rodent chow diet) and water. All procedures involving animals were performed in accordance with the ARRIVA guidelines,^13^ and ultrasonographic tumor detection/palpation was carried out according to license No. 113/2020, issued by the II Local Ethics Committee for Animal Experimentation (Kraków, Poland). Up to 9-month-old tumor-free KPC mice (males and females, former breeders included), or age/sex-matched control animals (LSL-Kras^+/+^; LSL-Trp53^+/+^; Pdx1-Cre; here, and thereafter, control-Cre), were humanely killed by cervical dislocation^14^ (for cytosolic Ca^2+^ measurements) or CO_2_ inhalation (other experiments). The pancreatic tissue was removed for further experimental procedures.

### Ultrasound Imaging

In our mouse model of pancreatic carcinogenesis (KPC mice), the presence of pancreatic abnormalities (eg, solid tumors) was examined by abdominal palpation on a weekly basis. In certain cases, KPC mice were also monitored by ultrasound imaging (USG) using a Vevo 2100 high-frequency, high-resolution digital imaging platform (VisualSonics, Canada), according to the previously described protocols.^15,16^

### Histology

Pancreatic tissues were fixed in formalin (48 h, RT), embedded in paraffin, and cut into 4 μm sections. The sections were then stained using our standard hematoxylin and eosin (HE) staining protocol,^17,18^ inspected under a light microscope DMi8 (Leica, Japan), photographed, and analyzed.

### Isolation of PACs and Cytosolic Ca^2+^ Measurements

Unless otherwise stated, extracellular buffer NaHEPES containing (m m) NaCl, 140; KCl, 4.7; HEPES, 10.0; MgCl_2_, 1.0, and glucose, 10.0 was supplemented with 1.0 m m Ca^2+^^19^ (in Ca^2+^ extrusion protocol: 10.0 m m Ca^2+^^14^). PACs were isolated as described previously^14^ from the pancreata of control-Cre mice, or tumor-free pancreatic tissues of KPC mice, and loaded with a Ca^2+^-sensitive fluorescent indicator Fluo-4 AM (5 μm; 30 min, RT). After loading, PACs were transferred to a flow chamber, and the experiments were performed in continuous perfusion with NaHEPES-based solutions. The cells were visualized using an LSM-880 AiryScan AxioVert confocal microscope equipped with an EC Plan-Neofluar 40×/1.30 Oil DIC M27 objective (Zeiss, Germany). Excitation was set to 488 nm laser light and emission to 593-630 nm. A series of images were recorded at 256 × 256 pixel resolution; two consecutive frames were averaged, and the time resolution was 1 image per 2 s. Fluorescence signals were plotted as F/F_0_, where F_0_ was an averaged signal from the first 10 baseline images, normalized as previously described.^20^

### Calcium Extrusion

For the Ca^2+^-extrusion experiments, PACs were pretreated with 2 μm thapsigargin (Tg) for 10 min in the absence of extracellular Ca^2+^. Next, again in Ca^2+^-free extracellular buffer, 10 μm Tg was applied for 200 s, and then the extracellular Ca^2+^ concentration was increased to 10 m m, which induced Ca^2+^ influx to the cytosol. Once a cytosolic plateau was reached, extracellular Ca^2+^ was reduced to 0 in the presence of 2 m m EGTA. This abrupt removal of extracellular Ca^2+^ unmasked Ca^2+^ extrusion across the plasma membrane. This phase of the response was further analyzed and compared between PACs isolated from control-Cre and KPC mice. For every recorded Ca^2+^ trace, the normalized fluorescence that corresponds to half the decrease between the maximum and minimum *F*/*F*_0_ values was calculated as follows: *F*_1/2_ = *F*_min_ + (*F*_max_ − *F*_min_)/2, according to the previously described methods.^14,21^ The time values corresponding to *F*_max_ (*t*_max_), and F_1/2_ (*t*_1/2_) were calculated from the linear fit to the extrusion phase. Finally, *t*_1/2_ was calculated as the difference between *t*(F_1/2_) and *t*_max_.

### Immunoblotting

KPC and control-Cre mouse pancreatic tissue samples were homogenized using a TissueLyser (Qiagen, USA), in RIPA buffer supplemented with a protein inhibitor cocktail. The protein concentration was assessed by the BCA assay. 15 μg protein samples were loaded into the NuPAGE 4%-12% Bis-Tris precast gels and resolved (180 V, 1 h) using SDS/PAGE electrophoresis in MOPS buffer. The overnight transfer (25 V) in a Mini Trans-Blot Cell (Bio-Rad, USA) was performed onto the 0.2 μm PVDF blotting membranes. Then the membranes were blocked in 5% non-fat dry milk in TBS supplemented with 0.05% Tween-20 (TBS-T). The membranes were incubated (1 h, RT) with primary antibodies: anti-calcium pump pan PMCA ATPase mouse monoclonal antibody (1:1000) or anti-vinculin mouse monoclonal antibody (1:5000), and then were washed with TBS-T (5 min; 3×). Next, the membranes were incubated (1 h, RT) with the secondary antibody: horse anti-mouse IgG Antibody (H + L), peroxidase (1:5000), and then were washed with TBS-T (5 min; 3×). Protein detection was performed in a ChemiDoc Imaging System (Bio-Rad, USA) using Immobilon Western Chemiluminescent HRP Substrate. The ImageLab software was used for the densitometric analysis.

### Cell Death and Viability Measurements

(1) For cell death assessments, PACs were stimulated for 2 h at RT with 60 μm Men (in ethanol stock; or equal volumes of ethanol) in extracellular NaHEPES buffer, containing 1 m m Ca^2+^ or Ca^2+^-free. One hour before the incubation end, the cells were stained with the NucView 488 & RedDot 2 Apoptosis and Necrosis Kit, which reports caspase-3/7 activity with a green fluorescence signal and the presence of dead cells with far-red fluorescence signal, according to the manufacturer’s instructions. PACs were transferred to a glass-bottom chamber and imaged using a DMI8 fluorescence microscope equipped with an HC PL APO 40x/1.30 OIL objective and a DFC7000GT camera (all: Leica, Japan). The following parameters were applied for NucView 488 imaging: excitation 490 nm, 5% illumination power, an FITC emission filter; and for RedDot 2 imaging: excitation 660 nm, 9% illumination power, and a CY5 emission filter. A total of 15 random images were collected, and live, apoptotic, and necrotic cells were counted, and their numbers were averaged and presented as percentages of the total ± SEM. (2) For the ATP-based cell viability measurements, a CellTiter-Glo 3D Cell Viability Assay was used to quantify ATP levels in acinar cells freshly isolated from KPC and control-Cre mice, following the manufacturer’s protocol. Briefly, equal volumes of extracellular NaHEPES buffer containing PACs and CellTiter-Glo 3D Reagent were transferred to a 96 Well White/Clear Bottom Plate, shaken for 5 min, and left for 25 min at RT to stabilize the samples. The luminescence signals were recorded using an Infinite M200 Plate Reader (Tecan, Switzerland), and the results were normalized post-assay to the protein levels assessed by the BCA assay run in a reference plate, using the same cell aliquot as for the assay.

### Cell Metabolism Measurements

Metabolic functions of acinar cells isolated from KPC and control mice were assessed using a Seahorse XF Cell Mito Stress Test Kit, according to the previously described protocols,^22,23^ with some modifications. Briefly, 8-well cell culture plates were thin-coated with Matrigel 12 h before the measurements. PACs were isolated on ice, transferred to the plates, and allowed to attach. Next, extracellular NaHEPES buffer was replaced with XF DMEM pH 7.4 medium, supplemented with 10 m m glucose, 1 m m pyruvate, and 2 m m glutamine. The cells were incubated for 15-30 min at 37°C in a CO_2_-free I5110 dry oven (Labnet, USA), and then a mitochondrial stress test was performed using a Seahorse Bioscience XF HS Mini Analyzer (Agilent, USA). The cells were injected with sequential fluxes of:^23^ 1 µg/mL oligomycin applied to inhibit ATP synthase and cause a rapid hyperpolarization of the mitochondrial membranes; 0.3 µm FCCP, an uncoupling agent of oxidative phosphorylation, which restores proton transport across the mitochondrial inner membrane; and 2 µg/mL mix of antimycin A and rotenone, an inhibitor of complexes I and III, and thus a blocker of mitochondrial phosphorylation. The results were normalized post-assay to the cell numbers calculated by counting cell nuclei stained with Hoechst 33258 and analyzed using the QuPath software.^24^ Basal respiration, ATP production, maximal respiration, proton leak, spare respiratory capacity, non-mitochondrial oxygen consumption, and coupling efficiency were calculated with normalized oxygen consumption rate (OCR) changes.

### Statistical Analysis

Quantitative results were presented as averaged/representative linear plots (Ca^2+^ traces), box and whisker plots (area under the curve, t_1/2_, relative protein expression, OCR) showing individual data points together with a median and/or a mean, 10 × 10 dot plots (response abundancies), or bar charts (cell death assay) showing a mean ± SEM. For statistical analysis, appropriate parametric or nonparametric tests were applied using the GraphPad Prism 8.0.1 software (detailed description in the figure legends), and the significance threshold was set at *P* < .05.

## Results

### Physiological Ca^2+^ Responses in PAC

**Figure 2.**
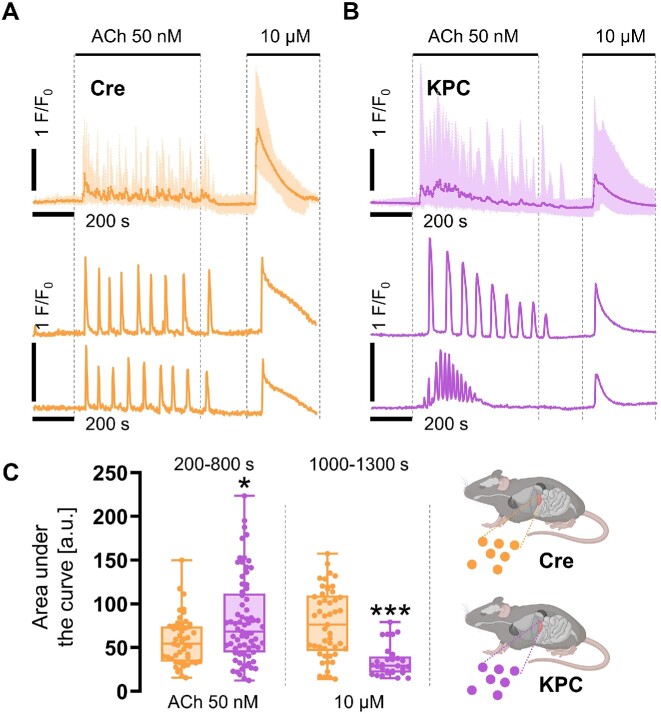
Responses of control-Cre and KPC pancreatic acinar cells to acetylcholine (ACh). (A and B) Cytosolic Ca^2+^ signals evoked in pancreatic acinar cells (PACs) by ACh at 50 n m and 10 μm concentrations. (A) Averaged Ca^2+^ traces, shown together with min to max values, and two representative [Ca^2+^]_i_ traces, recorded in PACs isolated from control-Cre mice; *N* = 5, *n* = 49. (B) Averaged Ca^2+^ traces, shown together with min to max values, and two representative [Ca^2+^]_i_ traces, recorded in PACs isolated from KPC mice; *N* = 7, *n* = 73. (C) Comparison of the area under the response curves evoked in PACs by ACh at 50 n m (*P < .05) and 10 μm (***P < .001) concentrations. Data presented as box and whisker plots, showing individual data points together with a median; orange: individual response areas of PACs isolated from control-Cre mice, *n* = 49; and purple: individual response areas of PACs isolated from KPC mice, *n* = 73. Statistical significance was assessed using the unpaired *t*-test, and the *P*-value < .05 was set as significant.

PACs were isolated from the pancreata of KPC mice (males and females, up to 9-month-old) that carry driver mutations in *Kras* (G12D) and *Trp53* (R172H), under the control of the Pdx1-Cre system, which promotes the development of PC. Control cells were obtained from Cre mice expressing the *Pdx1-Cre* construct, but in these animals, both alleles of *Kras* and *Trp53* genes were free from cancer-inducing point mutations. KPC and control-Cre PACs were isolated by collagenase digestion of the mouse pancreata, and they were essentially identical in terms of their morphological features. In order to characterize the capacity of KPC or control-Cre PACs to maintain physiological Ca^2+^ signals evoked by ACh, we first applied low 50 n m concentrations of the stimulant for 600 s and recorded Ca^2+^ responses induced in control-Cre (Figure 2A) and KPC PACs (Figure 2B). While cytosolic Ca^2+^ signals in control-Cre cells were characterized by a regular oscillatory pattern, that is, fine Ca^2+^ spikes with regular frequencies and similar amplitudes during the entire ACh stimulation (Figure 2A), in KPC cells, these oscillations evolved toward more global and sustained Ca^2+^ responses (Figure 2B). As evidenced by the individual [Ca^2+^]_i_ traces, not only were KPC acinar cells characterized by the initial presence of Ca^2+^ spikes at a higher frequency than in controls, but these spikes also had a higher signal amplitude (Figure 2A and B). During cell stimulation with 50 n m ACh, Ca^2+^ oscillations in KPC cells became gradually attenuated: both the frequency and amplitudes decreased (Figure 2B), but the responses were maintained longer in KPC PACs after removal of ACh at 800 s (Figure 2A and B). Analysis of the area under the response curve (measured between 200 and 800 s) revealed a significant increase (*P < .05) in the oscillatory responses recorded in KPC PACs compared to control-Cre acinar cells (Figure 2C), with exact mean values: 104.3 vs 63.2 [a.u.], respectively. After washing the cells with the extracellular buffer (supplemented with 1 m m Ca^2+^) for 200 s, PACs were treated with a supramaximal concentration of 10 μm ACh in order to trigger large responses. Global Ca^2+^ releases recorded in PACs isolated from control-Cre mice represented a typical single Ca^2+^ response characterized by a rapid Ca^2+^-release phase followed by a steady decline in cytosolic Ca^2+^ levels (Figure 2A). Similar response patterns were present in KPC PACs (Figure 2B), but in these cells, signal amplitudes were reduced. These differences between KPC and control-Cre cells were reflected by the significantly decreased (***P < .001) areas under the responses recorded in KPC vs control-Cre PACs (Figure 2C), with mean values 34.9 vs 89.7 [a.u.], respectively.

### Cytosolic Ca^2+^ Responses to Menadione

**Figure 3.**
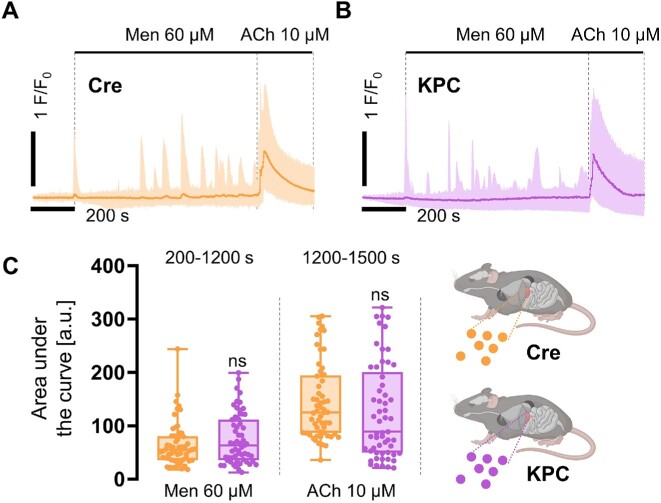
Intracellular Ca^2+^ signals elicited by menadione in control-Cre and KPC pancreatic acinar cells (PACs). (A and B) Averaged cytosolic Ca^2+^ signals evoked in pancreatic acinar cells (PACs) by 60 μm Men, and subsequent cellular responses to the supramaximal dose of acetylcholine (10 μm ACh), used as a positive control of cell viability. (A) Averaged Ca^2+^ traces, shown together with min to max values, recorded in PACs isolated from control-Cre mice; *N* = 6, *n* = 57. (B) Averaged Ca^2+^ traces, shown together with min to max values, recorded in PACs isolated from KPC mice; *N* = 6, *n* = 57. (C) Comparison of the area under the response curves evoked in PACs by 60 μm Men, and a subsequent application of 10 μm ACh concentrations. Data presented as box and whisker plots, showing individual data points together with a median; orange: individual response areas of PACs isolated from control-Cre mice, *N = 6, n* = 57; and purple: individual response areas of PACs isolated from KPC mice, *N = 6, n*  = 57. Statistical significance was assessed using the unpaired *t*-test, and the *P*-value < .05 was set as significant.

**Figure 4.**
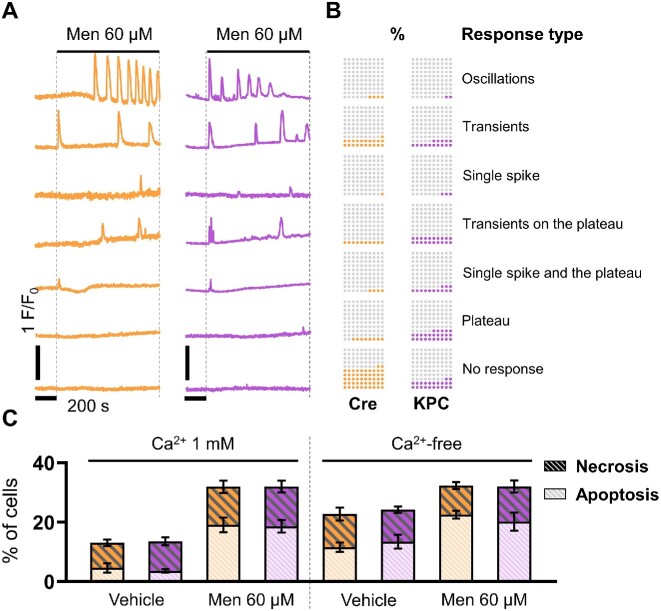
Patterns of the intracellular Ca^2+^ signals evoked by menadione in control-Cre and KPC pancreatic acinar cells. (A) Representative Ca^2+^ traces of the oscillatory, transient and plateau pattern, and the combinations of these, elicited in pancreatic acinar cells (PACs) by 60 μm menadione; orange: individual Ca^2+^ traces recorded in PACs isolated from control-Cre mice; and purple: individual Ca^2+^ traces recorded in PACs isolated from KPC mice. (B) 10 × 10 dot plots comparing the abundances of Ca^2+^ signals of the oscillatory, transient, and plateau pattern, and the combinations of these, elicited in PACs by 60 μm Men. Gray: lack of response; color: percentages of intracellular Ca^2+^ signals recorded in control-Cre (orange; *n* = 57) and KPC (purple; *n* = 57) pancreatic acinar cells. (C) Bar charts comparing levels of apoptosis (light gray dash pattern) and necrosis (dark gray dash pattern) in PACs induced by 60 μm Men, in the presence of 1 m m Ca^2+^, or in a Ca^2+^-free extracellular buffer. The results are shown as means ± SEM; *N* = 4.

Next, we compared the effects of Men on intracellular Ca^2+^ signals in KPC and Cre acinar cells. Historically used as a cost-effective substitute for vitamin K in fortified foods, Men evokes cytoplasmic Ca^2+^ elevations that take the form of oscillatory responses, Ca^2+^ transients, or Ca^2+^ plateaus (also combinations of all of the above, or no response). Owing to the presence of double bonds in its naphthoquinone ring, Men can initiate the production of reactive oxygen species in PACs. Cell stimulation with 60 μm Men elicited cytosolic Ca^2+^ responses in control-Cre (Figure 3A) and KPC PACs (Figure 3B). In both cell phenotypes, the averaged Ca^2+^ traces (Figure 3A and B) showed the presence of a Ca^2+^ peak immediately after Men application, and then the development of different Ca^2+^ responses, also on top of the elevated Ca^2+^ plateau. In KPC cells, Men evoked intracellular Ca^2+^ responses immediately after the initial peak (Figure 3B), while in control-Cre PACs, the responses were delayed by approximately 200 s (Figure 3A). This combination of spikes and transients resulted in an irregular pattern of Men-induced Ca^2+^ signals, maintained until the application of supramaximal concentrations of ACh, used as controls of the store loading (Figure 3A and B). As evidenced by the areas under the response curves to Men and ACh, no statistically significant differences were found between KPC and control-Cre cells (Figure 3C). While oscillations, transients, single spikes, and elevated Ca^2+^ plateaus were present both in KPC and in control-Cre cells, the proportions of distinct response types varied between these cells (Figure 4A and B). First, as many as 52% of control-Cre cells and only 22% of KPC PACs did not respond to Men (but responded to ACh). Furthermore, 58% of KPC cells developed one of the following responses characterized by the presence of an elevated Ca^2+^ plateau: transients on top of the prolonged plateau (20%), a single spike followed by the plateau (13%), and the plateau alone (25%). The corresponding values in control-Cre cells were much lower: 22% of the overall plateau-type responses, that is, 10% of transients on top of the plateau, 4% of a single spike followed by the plateau, and the plateau alone in 8% of total responses recorded in control-Cre cells (Figure 4B). In addition, the oscillatory type of responses, Ca^2+^ transients, and single Ca^2+^ spikes, also showed a different distribution in KPC and control-Cre cells: 2% vs 4% for the oscillatory responses, 15% vs 21% for transients, and 3% vs 1% for single Ca^2+^ spikes, respectively. We then compared apoptotic and necrotic death of KPC and control-Cre acinar cells incubated for 2 h with 60 μm Men in the presence of 1 m m Ca^2+^, or in a Ca^2+^-free extracellular buffer (Figure 4C). Independently of cell phenotype, Men induced apoptosis in approximately 20% of PACs, and removal of Ca^2+^ ions from the extracellular solution did not change the proportions of live, apoptotic, and necrotic cells (Figure 4C). Altogether, these data suggest that KPC PACs may have a decreased capacity to handle elevated levels of cytosolic Ca^2+^ compared to PACs isolated from control-Cre mice (Figure 4A and B). In the studied stage of onco-transformation, cell susceptibility and death in response to the noxious chemical stimuli were not altered in KPC cells compared to control-Cre cells (Figure 4C).

### Ca^2+^ Extrusion Across the Plasma Membrane

**Figure 5.**
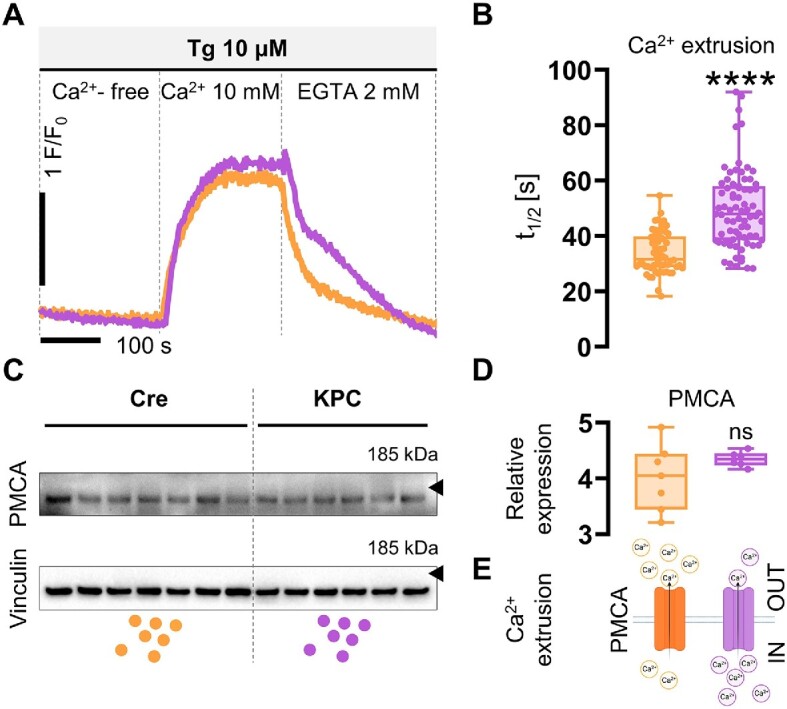
Comparison of Ca^2+^ extrusion rates across the cell membrane in control-Cre and KPC pancreatic acinar cells (PACs). (A) Representative Ca^2+^ traces recorded in PACs, treated first with 10 μm thapsigargin (Tg) in the absence of Ca^2+^ in extracellular buffer, then with 10 m m Ca^2+^ to achieve a formation of stabile Ca^2+^ plateau, and, subsequently, again in the absence of Ca^2+^ in extracellular buffer supplemented with 2 m m EGTA, to induce Ca^2+^ extrusion across the cell membrane. Orange: Ca^2+^ trace recorded in a PAC isolated from control-Cre control mice; *N* = 3, *n* = 58; and purple: Ca^2+^ trace recorded in a PAC isolated from KPC mice; *N* = 3, *n* = 82. (B) Comparison of the half-times (*t*_1/2_) of cytosolic Ca^2+^ extrusion across the cell membrane shows significantly slower (^****^P < .0001) extrusion rates in KPC acinar cells than in Cre controls. Ca^2+^ extrusion was induced in PACs by rapid removal of 10 m m Ca^2+^, subsequent application of 2 m m EGTA, and the continued presence of 10 μm Tg. Data presented as a box and whisker plot, showing individual data points together with a median; orange: individual *t*_1/2_ of PACs isolated from Pdx1-Cre mice, *n* = 58; and purple: individual *t*_1/2_ of PACs isolated from KPC mice, *n* = 82. Statistical significance was assessed using the Mann-Whitney U test, and the *P*-value < .05 was set as significant. (C) PMCA expression in pancreata isolated from control-Cre and KPC mice, *N* = 7 and *N* = 6, respectively. (D) Densitometric analysis revealed no significant differences in PMCA levels in control-Cre and KPC pancreatic samples. Data presented as a box and whisker plot, showing individual data points together with a median. (E) Schematic summary of data presented: although PMCA levels do not differ in control-Cre and KPC pancreatic acinar cells, Ca^2+^ extrusion rates through the cell membrane are slower in KPC cells, resulting in lower volumes of Ca^2+^ removed from the cytosol by PMCA.

In order to compare the speed of Ca^2+^ extrusion across the plasma membrane between control-Cre and KPC cells, we applied a previously published protocol (with modifications)^14,21^ (Figure 5A). We first incubated PACs in the Ca^2+^-free extracellular buffer supplemented with thapsigargin (Tg), a potent inhibitor of the endomembrane Ca^2+^ pump, sarco/endoplasmic reticulum Ca^2+^-ATPase (SERCA), to empty intracellular Ca^2+^ stores. The extracellular Ca^2+^ concentration was then changed to 10 m m, which caused an influx of Ca^2+^ into the cytosol (through the store-operated calcium entry [SOCE])—this was evidenced by the increase in cytosolic Ca^2+^ until a prolonged plateau was formed in both control-Cre and KPC cells (Figure 5A). Abrupt removal of extracellular Ca^2+^ and the addition of 2 m m EGTA quickly reduced cytosolic Ca^2+^ concentration to baseline levels, but the apparent rates were different in control-Cre and KPC cells (Figure 5A). The continuous presence of Tg in this phase of the experiment blocked SERCA from actively transporting Ca^2+^ to the ER. Therefore, under conditions of a Ca^2+^-free extracellular environment, the clearance of cytoplasmic Ca^2+^ from KPC and control-Cre cells toward basal Ca^2+^ concentrations reflected Ca^2+^ extrusion across the plasma membrane. Since PACs have only very minor (if any) sodium-calcium exchanger (NCX) activity,^25^ almost the entire Ca^2+^ extrusion is dependent on the plasma membrane Ca^2+^ ATPase (PMCA) in these cells.^21^ When extracellular Ca^2+^ was removed, KPC cells not only maintained a Ca^2+^ plateau longer than control-Cre cells but were also characterized by slower rates of Ca^2+^ extrusion than control PACs. The analysis of cytosolic Ca^2+^ clearance revealed a statistically significant difference (^****^P < .0001) in the PMCA-driven extrusion between KPC and control-Cre cells, here described as the time required for Ca^2+^ concentration to decrease by half the maximum amplitude (t_1/2_). The *t*_1/2_ values were much higher in KPC cells compared to control-Cre PACs: 47.91 vs 33.91 [s], respectively (Figure 5B). Importantly, the levels of PMCA expression in KPC and control-Cre acinar cells were comparable (Figure 5C and D). Taken together, in the studied KPC cells, cytosolic Ca^2+^ extrusion across the plasma membrane was slower than in control-Cre cells and this effect was not due to decreased expression of PMCA (Figure 5E).

### Mitochondrial Metabolism

**Figure 6.**
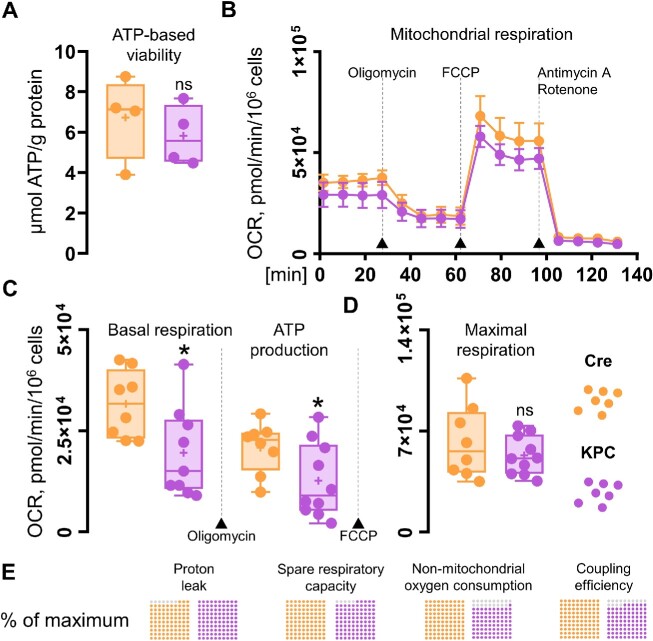
Comparison of cell metabolism in control-Cre and KPC pancreatic acinar cells. (A) Cell viability was assessed by comparing ATP production normalized to protein content in control-Cre (orange) and KPC (purple) pancreatic acinar cells (PACs); *N* = 4. (B) Mitochondrial respiration was analyzed in control-Cre (orange) and KPC (purple) PACs in response to 1 μg/mL oligomycin, 0.3 μm FCCP, and 2 μg/mL mix of antimycin A and rotenone. The results are shown as means ± SEM; *N* = 4, *n* = 8 control-Cre, and *n* = 10 KPC replicates. (C) Comparison of basal respiration (*P < .05) and ATP production (*P < .05) in control-Cre (orange) and KPC (purple) PACs. Data presented as a box and whisker plot, showing individual data points together with a median and a mean. Statistical significance was assessed using the unpaired *t-*test, and the *P*-value < .05 was set as significant. One outlier was removed from the analysis of basal respiration (the Grubbs test, α = .05). (D) Comparison of maximal respiration in control-Cre (orange) and KPC (purple) PACs. Data presented as boxes and whiskers, showing individual data points together with a median and a mean. Statistical significance was assessed using the unpaired *t*-test, and the *P*-value < .05 was set as significant. (E) 10 × 10 dot plots comparing proton leak, spare respiratory capacity, non-mitochondrial oxygen consumption, and coupling efficiency in PACs normalized to the maximal averaged value measured (100%). Gray: difference to 100%; color: percentages of the maximal parameter values detected in control-Cre (orange) and KPC (purple) PACs.

Aiming to identify the causes of significantly lower Ca^2+^ extrusion rates found in KPC acinar cells compared to control-Cre PACs, we investigated mitochondrial respiration in these cells (Figure 6). While the ATP-based cell viability assay did not show prominent differences between KPC and control-Cre cells (Figure 6A), a Seahorse XF Cell Mito Stress Test revealed that the basal respiration and ATP production were decreased (both *P < .05) in KPC cells compared to normal PACs (Figure 6B and C). Other parameters measured (mean values for control-Cre vs KPC), that is, the maximal respiration (62249.52 vs 53 144.53 [pmol/min/10^6^ cells]), spare respiratory capacity (30598.66 vs 28917.73 [pmol/min/10^6^ cells]), non-mitochondrial oxygen consumption (6009.10 vs 4867.59 [pmol/min/10^6^ cells]), proton leak (10 845.15 vs 11 641.88 [pmol/min/10^6^ cells]), and coupling efficiency (66 vs 57 [%]) were not markedly altered in KPC cells (Figure 6D and E).

## Discussion

Secretory acinar epithelia may undergo a phenotypic transformation into duct-like cellular structures, which, under the condition of cell stress and the presence of driver mutations in genes that encode Kras and Trp53 proteins, contribute toward pancreatic carcinogenesis (Figure 1). Here we studied whether cellular expression of Kras^G12D^ and Trp53^R172H^ in mouse PACs changes cell physiology and functions, investigated by analyses of cytosolic Ca^2+^ signals (Figures 2-4), the capacity for maintaining energy-dependent ion transport across the cell membrane (Figure 5), and mitochondrial respiration (Figure 6). First, we showed that KPC cells are characterized by the presence of physiological Ca^2+^ oscillations in response to 50 n m ACh, of higher amplitudes and frequencies than the oscillatory responses recorded in normal cells (Figure 2A and B). Such a pattern resulted in a greater (*P < .05) area under the response curve to 50 n m ACh, but a significantly lower (***P < .001) area under the transient-type responses to supramaximal 10 μm doses of ACh in PACs isolated from KPC mice compared to control-Cre cells (Figure 2C). Although the average responses to 60 μm Men were not significantly different between KPC and control-Cre cells (Figure 3), the proportions of distinct response types varied in those two cell phenotypes (Figure 4A and B). While PACs isolated from KPC mice had more plateau-type responses (Figure 4A and B), these did not translate into increased cell death of KPC cells treated with 60 μm Men, both in the presence of 1 m m Ca^2+^ or in the absence of Ca^2+^ in the extracellular solution (Figure 4C). James et al. compared the capacities for SOCE measured in human colorectal cancer HCT 116 cells expressing oncogenic KRAS^G13D^ and their isogenic controls (HKH-2 cells), wherein the mutant *KRAS* allele was deleted owing to the homologous recombination.^26^ Colorectal cancer cells were found to have SOCE repressed, and that protected these mutant cells from the apoptotic stimuli and cell death.^26^ Active removal of Ca^2+^ from the cytosol to the extracellular environment across the biological membranes is essential for maintaining low resting cytosolic Ca^2+^ levels.^9,27^ Processes of Ca^2+^ extrusion by Ca^2+^ pumps require constitutive hydrolysis of ATP, but this is the metabolic “price to pay” in order to prevent accumulation (overload) of Ca^2+^ in the cytosol and cell death.^9,28^ Notably, sustained elevations in cytosolic Ca^2+^ reduce ATP synthesis.^29^ Since KPC acinar cells were characterized by significantly lower rates of Ca^2+^ extrusion (^****^P < .0001) than control-Cre PACs (Figure 5B), and PMCA expression levels did not differ in these cells (Figure 5C and D), we analyzed the mitochondrial respiration (Figure 6). Production of ATP in mitochondria is vital for cellular homeostasis, since this requires energy-consuming processes, for example, molecule transport against the chemical gradient.^9,28^ We compared mitochondrial respiration in KPC and control-Cre PACs (Figure 6B) and found that the basal respiration was decreased (*P < .05), and so was the production of ATP (*P < .05) in PACs isolated from KPC mice (Figure 6C). Of note is that PACs isolated from these mice were a mix of the normal, premalignant, and malignantly transformed clones, owing to the stochastic expression of Cre recombinase in pancreatic tissue.^12^ Other parameters of cell metabolism, for example, the maximal respiratory capacity (Figure 6D), the spare respiratory capacity, and non-mitochondrial oxygen consumption (Figure 6E), were not significantly different in KPC and control-Cre PACs. Previous studies reported that PC cell lines PANC-1 and MIA PaCa-2 had their PMCA activities strongly dependent on glycolytic ATP production, and not on the mitochondrial phosphorylation.^30,31^ As such, the inhibition of glycolysis in these cells caused a marked ATP depletion, inhibition of PMCA, and cytosolic Ca^2+^ overload.^30^ Here, using freshly isolated KPC acinar cells, we show that prior to undergoing a completed oncogenic transformation and acquiring the malignant phenotype, epithelial precursors of PC have impaired Ca^2+^ extrusion (Figure 5A and B). Although we found that the observed effect was mainly dependent on mitochondrial respiration (Figure 6B and C), and likely not glycolysis (Figure 6E), it is possible that in the course of pancreatic carcinogenesis, in which metabolism shifts from aerobic toward glycolytic, PMCA may also become dependent on the anaerobic source of ATP. Because of the genetic design of KPC mice,^2,12^ their Pdx1-expressing pancreatic cells carry a point mutation in the *Kras* gene that yields the constitutively active GTPase Kras^G12D^, which requires binding the GTP molecule for signal transduction.^32^ Even though ATP serves as the main molecule for energy conservation in the cells, GTP is required for energy transfer in the tricarboxylic acid cycle.^28^ Since Kras^G12D^ constitutively hydrolyzes GTP into GDP, this further depletes energy pools available in mutant cells. Taken together, even before the malignant KPC phenotype becomes apparent, mutant epithelia not only have a lower capacity for ATP production (*P < .05) in mitochondrial respiration (Figure 6), but also hydrolyze GTP molecules by Kras^G12D^. This, in turn, decreases their capacity for fine-tuning the compartmental Ca^2+^ concentration, resulting in a less regular pattern of the cytosolic Ca^2+^ signals than in control-Cre acinar cells (Figures 2 and 4).

## Data Availability

Data will be made available upon reasonable request to the corresponding author.

## References

[1] Siegel RL , Miller KD, Fuchs HE, Jemal A. Cancer statistics, 2021. CA Cancer J Clin. 2021;71(1):7–33. 33433946 10.3322/caac.21654

[2] Hingorani SR , Petricoin EF, Maitra A, et al. Preinvasive and invasive ductal pancreatic cancer and its early detection in the mouse. Cancer Cell. 2003;4(6):437–450. 14706336 10.1016/s1535-6108(03)00309-x

[3] Hill W , Zaragkoulias A, Salvador-Barbero B, et al. EPHA2-dependent outcompetition of KRASG12D mutant cells by wild-type neighbors in the adult pancreas. Curr Biol. 2021;31(12):2550–2560.e5. 33891893 10.1016/j.cub.2021.03.094 PMC8231095

[4] Crawford HC . Putting the cell of origin for pancreatic cancer into its proper context. Cell Mol Gastroenterol Hepatol. 2019;8(4):645–646. 31473247 10.1016/j.jcmgh.2019.08.001 PMC6889687

[5] Storz P . Acinar cell plasticity and development of pancreatic ductal adenocarcinoma. Nat Rev Gastroenterol Hepatol. 2017;14(5):296–304. 28270694 10.1038/nrgastro.2017.12 PMC6036907

[6] Farrell AS , Joly MM, Allen-Petersen BL, et al. MYC regulates ductal-neuroendocrine lineage plasticity in pancreatic ductal adenocarcinoma associated with poor outcome and chemoresistance. Nat Commun. 2017;8(1):1728. 29170413 10.1038/s41467-017-01967-6 PMC5701042

[7] Gärtner S , Krüger J, Aghdassi AA, et al. Nutrition in pancreatic cancer: a review. Gastrointest Tumors. 2016;2(4):195–202. 27403414 10.1159/000442873 PMC4924449

[8] Argent BE , Gray MA, Steward MC, Case RM. Cell physiology of pancreatic ducts. In: Johnson LR, ed. Physiology of the Gastrointestinal Tract. 4th ed. London: Academic Press. 2006:1371–1396.

[9] Petersen OH . Watching living cells in action in the exocrine pancreas: the Palade Prize Lecture. Function. 2022;4(1):zqac061. 10.1093/function/zqac061 36606242 PMC9809903

[10] Petersen OH , Gerasimenko JV, Gerasimenko OV, Gryshchenko O, Peng S. The roles of calcium and ATP in the physiology and pathology of the exocrine pancreas. Physiol Rev. 2021;101(4):1691–1744. 33949875 10.1152/physrev.00003.2021

[11] Pérez S , Pereda J, Sabater L, Sastre J. Redox signaling in acute pancreatitis. Redox Biol. 2015;5:1–14. 25778551 10.1016/j.redox.2015.01.014 PMC4360040

[12] Hingorani SR , Wang L, Multani AS, et al. Trp53R172H and KrasG12D cooperate to promote chromosomal instability and widely metastatic pancreatic ductal adenocarcinoma in mice. Cancer Cell. 2005;7(5):469–483. 15894267 10.1016/j.ccr.2005.04.023

[13] SN du Percie , Hurst V, Ahluwalia A, et al. The ARRIVE guidelines 2.0: updated guidelines for reporting animal research. PLoS Biol. 2020;18(7):e3000410. 32663219 10.1371/journal.pbio.3000410 PMC7360023

[14] Jakubowska MA , Kerkhofs M, Martines C, et al. ABT-199 (Venetoclax), a BH3-mimetic Bcl-2 inhibitor, does not cause Ca^2+^-signalling dysregulation or toxicity in pancreatic acinar cells. Br J Pharmacol. 2019;176(22):4402–4415. 30266036 10.1111/bph.14505 PMC6887725

[15] Sastra SA , Olive KP. Quantification of murine pancreatic tumors by high-resolution ultrasound. Methods Mol Biol. 2013;980:249–266. 23359158 10.1007/978-1-62703-287-2_13 PMC3879959

[16] Jakubowska MA , Pyka J, Michalczyk-Wetula D, et al. Electron paramagnetic resonance spectroscopy reveals alterations in the redox state of endogenous copper and iron complexes in photodynamic stress-induced ischemic mouse liver. Redox Biol. 2020;34:101566. 32464500 10.1016/j.redox.2020.101566 PMC7251382

[17] Jakubowska M , Sniegocka M, Podgorska E, et al. Pulmonary metastases of the A549-derived lung adenocarcinoma tumors growing in nude mice. A multiple case study. Acta Biochim Pol. 2013;60(3):323–330. 23828777

[18] Kusiak AA , Jakubowska MA, Stopa KB, et al. Activation of pancreatic stellate cells attenuates intracellular Ca^2+^ signals due to downregulation of TRPA1 and protects against cell death induced by alcohol metabolites. Cell Death Dis. 2022;13(8):744. 36038551 10.1038/s41419-022-05186-w PMC9421659

[19] Jakubowska MA , Ferdek PE, Gerasimenko OV, Gerasimenko JV, Petersen OH. Nitric oxide signals are interlinked with calcium signals in normal pancreatic stellate cells upon oxidative stress and inflammation. Open Biol. 2016;6(8):160149. 27488376 10.1098/rsob.160149 PMC5008014

[20] Chvanov M , Gerasimenko OV, Petersen OH, Tepikin AV. Calcium-dependent release of NO from intracellular S-nitrosothiols. EMBO J. 2006;25(13):3024–3032. 16810320 10.1038/sj.emboj.7601207 PMC1500983

[21] Ferdek PE , Gerasimenko JV, Peng S, Tepikin AV, Petersen OH, Gerasimenko OV. A novel role for Bcl-2 in regulation of cellular calcium extrusion. Curr Biol. 2012;22(13):1241–1246. 22704985 10.1016/j.cub.2012.05.002 PMC3396842

[22] Armstrong JA , Sutton R, Criddle DN. Pancreatic acinar cell preparation for oxygen consumption and lactate production analysis. Bio Protoc. 2020;10(10):e3627. 10.21769/BioProtoc.3627 PMC7842544 33659300

[23] Gu X , Ma Y, Liu Y, Wan Q. Measurement of mitochondrial respiration in adherent cells by Seahorse XF96 cell Mito stress Test. STAR Protocols.2021;2(1):100245. 33458707 10.1016/j.xpro.2020.100245 PMC7797920

[24] Bankhead P , Loughrey MB, Fernández JA, et al. QuPath: open source software for digital pathology image analysis. Sci Rep. 2017;7(1):16878. 29203879 10.1038/s41598-017-17204-5 PMC5715110

[25] Petersen OH Localization and regulation of Ca2+ entry and exit pathways in exocrine gland cells. Cell Calcium. 2003;33(5):337–344. 12765680 10.1016/s0143-4160(03)00047-2

[26] Pierro C , Zhang X, Kankeu C, Trebak M, Bootman MD, Roderick HL. Oncogenic KRAS suppresses store-operated Ca^2+^ entry and I(CRAC) through ERK pathway-dependent remodelling of STIM expression in colorectal cancer cell lines. Cell Calcium. 2018;72:70–80. 29748135 10.1016/j.ceca.2018.03.002 PMC6291847

[27] Bruce JIE . Metabolic regulation of the PMCA: role in cell death and survival. Cell Calcium. 2018;69:28–36. 28625348 10.1016/j.ceca.2017.06.001 PMC5761718

[28] Plattner H , Verkhratsky A. Inseparable tandem: evolution chooses ATP and Ca^2+^to control life, death and cellular signalling. Philos Trans R Soc Lond B Biol Sci. 2016;371(1700):20150419. 27377729 10.1098/rstb.2015.0419 PMC4938020

[29] Peng S , Gerasimenko JV, Tsugorka T, et al. Calcium and adenosine triphosphate control of cellular pathology: asparaginase-induced pancreatitis elicited via protease-activated receptor 2. Philos Trans R Soc Lond B Biol Sci. 2016;371(1700):20150423. 27377732 10.1098/rstb.2015.0423 PMC4938023

[30] James AD , Chan A, Erice O, Siriwardena AK, Bruce JI. Glycolytic ATP fuels the plasma membrane calcium pump critical for pancreatic cancer cell survival. J Biol Chem. 2013;288(50):36007–36019. 24158437 10.1074/jbc.M113.502948 PMC3861649

[31] James AD , Patel W, Butt Z, et al. The plasma membrane calcium pump in pancreatic cancer cells exhibiting the Warburg effect relies on glycolytic ATP*. J Biol Chem. 2015;290(41):24760–24771. 26294767 10.1074/jbc.M115.668707 PMC4598988

[32] Biancur DE , Kimmelman AC. The plasticity of pancreatic cancer metabolism in tumor progression and therapeutic resistance. Biochim Biophys Acta Rev Cancer. 2018;1870(1):67–75. 29702208 10.1016/j.bbcan.2018.04.011 PMC6345383

